# Effects of smartphone use on postural control and gait performance in older women with neck pain

**DOI:** 10.1186/s12877-026-07831-x

**Published:** 2026-06-22

**Authors:** Bhiromlak Charoenrat, Somporn Sungkarat, Chatchai Phirawatthakul, Sureeporn Uthaikhup

**Affiliations:** Department of Physical Therapy, Integrated Neuro-Musculoskeletal, Chronic Disease, and Aging Research Engagement Center (ICARE), Faculty of Associated Medical Sciences, Chiang Mai University, Chiang Mai, 50200 Thailand

**Keywords:** Balance, Fall prevention, Gait, Mobility, Neck pain, Older adults, Smartphone

## Abstract

**Background:**

Neck pain is associated with altered postural and gait performance in older adults. Smartphone use may further challenge postural and gait control due to additional cognitive and motor demands. This study aimed to investigate the effects of smartphone use on postural control and gait performance in older adults with neck pain.

**Methods:**

Sixty-eight older women (34 with neck pain and 34 controls) were recruited. Postural control was assessed using a force plate under three conditions: standing with no smartphone use (S), standing while texting (S-text), and standing while talking (S-talk). Gait performance was assessed using a three-dimensional motion capture system under three conditions: walking with no phone use (W), walking while watching (W-watch), and walking while talking (W-talk). Postural outcomes included sway displacement in antero-posterior (AP) and medio-lateral (ML) directions, sway area, and sway velocity. Gait parameters included speed, cadence, stance and swing times, stride and step lengths, and step width. Absolute dual-task performance cost (abs-DTC) was calculated for all outcomes.

**Results:**

No significant interactions between group and condition were observed for any postural sway and gait outcomes (*p* > 0.05). Significant main effects of group and condition were found for all outcomes (*p* < 0.05). Regardless of condition, the neck pain group showed greater sway displacements (AP and ML), sway area, and velocity during standing compared with controls (*p* < 0.05). The neck pain group demonstrated reduced gait speed, lower cadence, longer stance and swing times, shorter stride and step lengths, and larger step width compared with controls (*p* < 0.05). Abs-DTC values were higher in the neck pain group than controls for all postural and gait outcomes (*p* < 0.05). Regardless of group, greater postural sway and gait impairments were generally observed during the talking condition compared with the other conditions (*p* < 0.05).

**Conclusion:**

Older women with neck pain demonstrated greater impaired postural control and gait across all conditions, including smartphone dual-task conditions, compared with controls. No significant group-by-condition interaction was observed, suggesting comparable effects of smartphone use across groups. Talking on a smartphone resulted in greater impairments than texting or watching across groups. These findings suggest the importance of increasing awareness of smartphone use in older women.

## Introduction

Neck pain has garnered increasing attention in both research and clinical practice due to its association with impaired postural control and gait, particularly among older adults. Altered cervical afferent input is postulated to cause sensory mismatches within the central nervous system and disrupt multisensory integration, leading to postural and gait instability [1, 2]. Evidence suggests that older adults with neck pain demonstrate impairments in standing balance and gait beyond those expected from normal aging [3–6]. The impairments include increased sway in both anteroposterior (AP) and mediolateral (ML) directions, larger sway area and higher sway velocity during standing [3, 4], as well as reduced gait speed, shorter stride length, and lower cadence during walking [4–6]. In addition, the impairments are evident under neck torsion, head movement, and dual-task conditions [3–7]. Previous studies have demonstrated that older adults with chronic neck pain exhibit greater postural sway during standing in a neck-torsion position, slower gait speed, lower cadence and longer gait cycle duration during walking with head turns [3–6], as well as greater difficulty walking while carrying a tray with glasses and performing a digit span task compared to those without neck pain [7]. A recent study has also shown that older adults who experienced at least one fall during a 1-year follow-up period were associated with reduced neck mobility [8]. Thus, impaired balance and gait performance, particularly under additional cognitive and motor demands may further increase risk of falls and compromise the ability to perform daily activities safely and independently in older adults with neck pain.

Smartphones have increased convenience in daily life and their use during everyday activities, including standing and walking, is common. However, activities such as texting, watching or talking on a smartphone while standing and walking impose dual-tasking demands that divert attention and interfere with sensory integration, leading to increased postural and gait instability [9–11]. A study demonstrated that mobile phone use (i.e., talking and dialing) significantly impaired standing balance in both younger and older adults, with the effects being more pronounced during talking than dialing [12]. The dial condition was also found to be associated with greater declines in mobility in older adults [12]. Additionally, several studies have shown that mobile phone use (e.g., looking, texting, and answering) altered gait patterns in older adults, including decreases in gait speed, stride length, and step length as well as increases in step time and cadence [9, 13, 14].

Given the association between neck pain and impairments in balance and gait, investigating smartphone use during daily activities (i.e., standing and walking) in older adults with neck pain is warranted. However, this issue has not yet been explored in this population. Smartphone use may compound existing postural and gait deficits through combined alterations in cervical afferent input and dual-task interference, thereby influencing postural control and spatiotemporal gait parameters and potentially increasing the risk of falls in this population. Thus, the aim of this study was to examine the effects of smartphone use on postural control and gait performance in older adults with neck pain. The study included only women to account for the higher prevalence of neck pain among females, consistent with sex-stratified global burden estimates [15]. It was hypothesized that older adults with neck pain would exhibit greater impairments in postural control during standing and gait while using a smartphone compared to healthy controls.

## Subjects and methods

### Participants

The sample size for the study was calculated using G*Power 3.1 for a mixed-model ANOVA design, assuming a medium effect size (Cohen’s f = 0.25), which represents the conventional standard balancing the detectability of realistic effects with feasible sample sizes [16], power of 0.80, and a significance level of 0.05. For model 1 (2 groups [neck pain vs control] × 3 conditions [single and two dual-task conditions]), a total sample of 56 participants (28 per group) was required. For model 2 (2 groups [neck pain vs control] × 2 conditions [dual-task cost comparisons]), a total of 68 participants (34 per group) was required. The larger estimated sample size was chosen to ensure adequate statistical power for all planned analyses.

Sixty-eight women aged 60 to 75 years (34 with neck pain and 34 controls) were recruited through advertisements in hospitals, physical therapy clinics, communities, and/or social media platforms such as Facebook and Line. Age and body mass index (BMI) were similar between the two groups. The neck pain group had non-specific neck pain (i.e., no specific underlying pathoanatomical cause or identifiable pathology) for at least three months [17], an average pain intensity of ≥ 3 on a 0–10 Numerical Rating Scale (NRS) over the past week [18] and a score of ≥ 10/100 on the Neck Disability Index (NDI) [19]. The control group had no history of neck pain for the past year. All participants were required to own a smartphone and be able to independently use and type on the device.

The exclusion criteria for both groups included a history of neck or head injury or surgery, recurrent headaches or dizziness, vestibular disorders (e.g., benign paroxysmal positional vertigo or Meniere’s disease); neurological or musculoskeletal conditions affecting balance and gait (e.g., myofascial pain, inflammatory joint disease, spinal/lower limb pain, deformities, or fractures, and symptoms of peripheral nerve involvement); neurocognitive disorders (e.g., dementia and Parkinson’s disease); uncorrected visual impairments; use of four or more medications [20]; medications known to affect balance and gait; and inability to walk independently or with walking aids. These conditions were screened through both subjective history-taking and objective clinical assessments.

### Standing balance assessment

A computerized force plate system (Model BTS P-6000; BTS Bioengineering Corporation, Quincy, MA) with a sensitive area of 40 × 60 cm was used to assess postural sway during standing balance tests at a sampling frequency of 500 Hz and low-pass filtered at 10 Hz using a fourth-order zero-phase Butterworth filter. The system provides fully digital signal output and measures forces up to ± 8000 N along the X, Y, and Z axes. Foot position was controlled using landmarks on the force plate surface, with compliance ensured through standardized verbal instructions. Standing balance was assessed barefoot in a comfortable stance with feet positioned shoulder-width apart under three conditions: standing without smartphone use (S), standing while texting on a smartphone (S-text), and standing while talking on a smartphone (S-talk). These positions reflect everyday situations that challenge postural control differently: S as baseline, S-text adding visual and motor distraction, and S-talk adding cognitive and auditory distraction. The S condition was performed first, followed by the S-text and S-talk conditions in randomized order using a random number table to minimize potential order effects. During the S condition, participants were instructed to stand still with their head facing forward and arms relaxed at their sides. In the S-text condition, participants were asked to use both hands to type responses on their smartphones to answer questions, such as date of birth, previous occupation, and address. During the S-talk condition, participants verbally responded to personal questions, such as “Tell me about your hobbies?” while an examiner remained in a separate room. Each condition was measured three times, with each trial lasting 30 s. A three-minute rest interval was provided between conditions to reduce fatigue and ensure reliable postural sway measurements.

Postural sway was analyzed using the SMART-Clinic software (BTS Bioengineering Corporation, Quincy, MA). Sway parameters included anterior–posterior (AP) displacement, medial–lateral (ML) displacement, sway area, and sway velocity. The software processes force plate data to calculate two-dimensional CoP trajectories, enabling quantitative assessment of postural control by capturing the magnitude and velocity of body sway in the AP and ML directions, as well as the overall sway area during standing balance tasks.

### Gait assessment

**Fig. 1:**
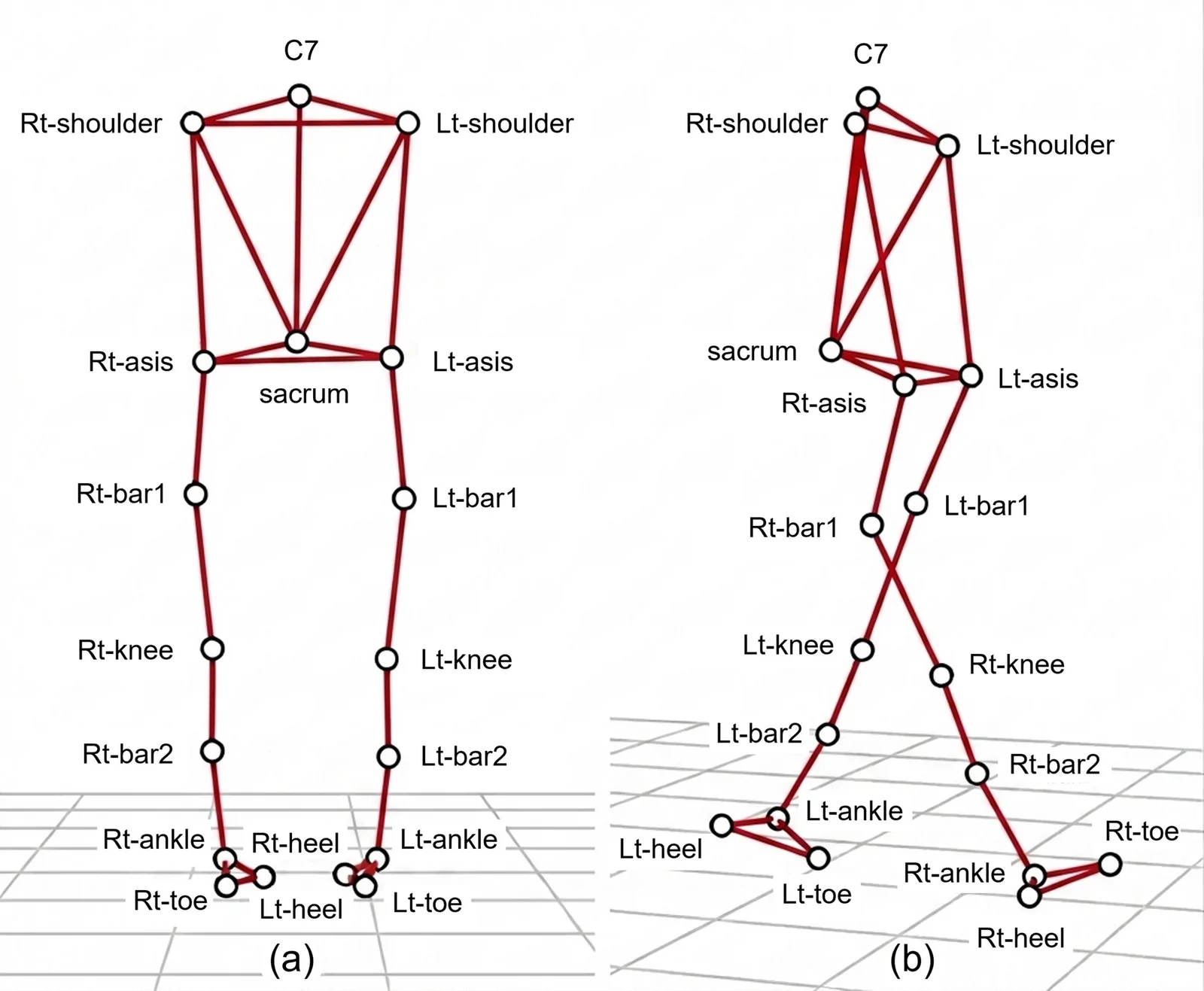
Placements of reflective markers Lateral view: Lt = left side; Rt = right side; C7 = 7th cervical vertebra; shoulder = lateral part of the acromion; asis = anterior superior iliac spine; sacrum = second sacral vertebra; bar 1 = lateral bar of the thigh; knee = lateral aspect of the knee flexion–extension axis; bar 2 = lateral bar of the shank; toe = space between the heads of the second and third metatarsals; ankle = lateral malleolus; heel = heel at the same height to toe

An eight-camera three-dimensional (3D) motion capture system (SMART-DX Motion Capture, MI, Italy) was used to collect gait parameters at a sampling rate of 250 Hz. Marker trajectories were filtered using a low-pass filter with a cutoff frequency of 25 Hz. Participants wore standardized study clothing provided by the examiner. The reflective markers were then attached to specific anatomical landmarks on the pelvis, thighs, and shanks/feet in accordance with the Helen Hayes protocol [21], with additional C7 and bilateral acromion markers to capture sagittal posture and trunk acceleration during gait (Fig. 1). Participants were asked to walk barefoot over a 10-m walkway under three conditions: usual walking without smartphone use (W), walking while watching a smartphone screen (W-watch), and walking while talking on a smartphone (W-talk). These conditions represent everyday walking scenarios: Was baseline, W-watch adding visual distraction, and W-talk adding cognitive distraction. The W condition was performed first, followed by the W-watch and W-talk conditions in a randomized order using a random number table to minimize potential order effects. During the W condition, participants were instructed to walk at a comfortable speed while looking straight ahead. In the W-watch condition, participants were instructed to hold their smartphones with both hands and watched a standardized video of neutral content (e.g., nature scenery without narrative audio) on the screen. The holding position was self-selected to reflect naturalistic smartphone use. In the W-talk condition, participants were asked to retrieve the phone from an examiner, who was located in a separate room, and respond to personal questions, such as “Talk about your daily routine”, while walking. Each condition was performed three times, and a three-minute rest interval was provided between conditions. One practice trial of usual walking was allowed for familiarization with the test. Gait was measured over the central 6-m of a 10-m walkway, excluding the initial 2 m for acceleration and the final 2 m for deceleration.

Data acquisition and processing were managed using SMART-Clinic software (BTS Bioengineering, MI, Italy), integrated with the SMART-DX motion system. Gait parameters included gait speed (m/s), cadence (steps/min), stance time (from initial contact to toe-off of the same foot), swing time (from toe-off to initial contact of the same foot), stride length (distance between two consecutive initial contacts of the same foot), step length (distance between initial contacts of opposite feet), and step width (lateral distance between consecutive heel strikes of opposite feet).

### Study procedure

**Fig. 2:**
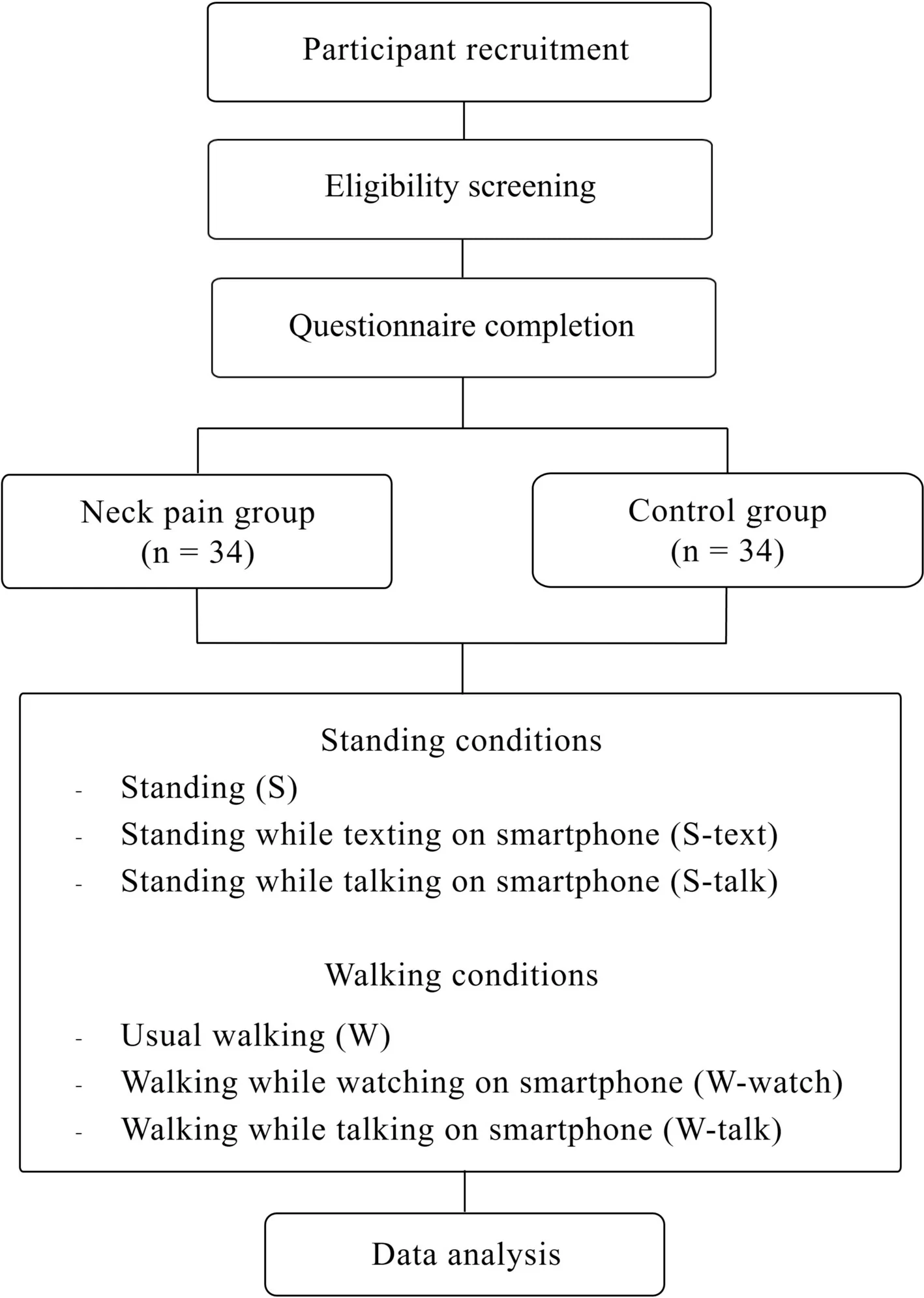
Flowchart of the study procedure

Potential participants underwent initial eligibility screening via telephone interview conducted by an independent researcher. All eligible participants were instructed to refrain from using any medications that could influence balance for at least six hours before the testing. On the test day, participants completed study questionnaires, including a general questionnaire designed to collect demographic data and information regarding smartphone use and neck pain. Participants with neck pain also completed the NDI to evaluate the impact of neck pain on daily activities [19]. Standing balance assessment was first performed, followed by gait assessment, with a 10-min rest between the two assessments. The examiners who performed all balance and gait assessments were blinded to the participants’ group allocation. The assessments were conducted in a quiet room. The flowchart of the study procedure is presented in Fig. 2.

### Data management and statistical analysis

The mean values of the three trials for each condition in the standing and gait assessments were used for analysis. Dual-task performance costs (DTCs) were also calculated as the percentage change between single-task performance (S or W condition) and dual-task performance (S-text, S-talk, W-watch, or W-talk condition) using the following formula:

$$\begin{aligned}&\text{Dual-task performance cost (DTC)}\\&=\frac{\text{single task performance - } \text{dual-task performance}}{\text{single task performance}}\times 100\end{aligned}$$

For variables in which higher mean values reflect poorer performance (e.g., sway displacement, sway area, sway velocity, stance and swing times, and step width), more negative DTC values reflect greater decrement under the secondary task (phone use). For variables where higher values indicate better performance (e.g., stride and step lengths, gait speed, and cadence), more positive DTC values indicate greater decrement under the secondary task. Subsequently, absolute dual-task performance cost (abs-DTC) values, irrespective of direction were reported, which smaller values were interpreted as better dual-task ability.

Independent t-test and chi-square were used to determine differences in demographic data between groups. Data normality was assessed using the Shapiro–Wilk test. As most variables were non-normally distributed, log10 transformation was applied to reduce skewness and improve variance homogeneity. For analytical consistency, the transformation was applied to all variables. A mixed-model ANOVA was used to analyze within- and between- group differences in log10-transformed mean values and log10-transformed abs-DTC values of all postural sway and gait outcomes. P-values were reported as uncorrected when the assumption of sphericity was met (Mauchly’s test *p* > 0.05) and were adjusted using the Greenhouse–Geisser correction when sphericity was violated (Mauchly’s test *p* ≤ 0.05) [22]. When main or interaction effects were identified, Bonferroni post hoc tests were performed for multiple comparisons. Effect sizes were calculated using partial eta squared (${\eta}_{P}^{2}$) and interpreted as follows: 0.01–0.05 (small), 0.06–0.13 (medium), and > 0.14 (large) [16]. The significance level was set at *p* < 0.05.

## Results

### Participants

There were no significant differences in demographic data between groups (*p* > 0.05, Table 1).

**Table 1:** Demographic data and characteristics of neck pain

		**Neck pain**	**Controls**
		**(*n* = 34)**	**(*n* = 34)**
Demographic data			
Age (years)		66.0 ± 4.0	68.1 ± 4.6
Height (m)		1.6 ± 0.1	1.5 ± 0.1
Weight (kg)		55.9 ± 9.9	55.1 ± 8.5
Faller/non-faller (n)		7/27	5/29
Medical conditions (n)			
- Hypertension		10	9
- Diabetes mellitus		3	2
Time spent using a smartphone (hours/day)		4.6 ± 3.0	4.6 ± 3.1
Characteristics of neck pain			
Intensity (NRS: 0–10)		5.2 ± 1.5	-
Disability (NDI: 0–100)		21.4 ± 7.9	-
Duration (years)		3.9 ± 4.1	-
Sides of neck pain (n)			
- Unilateral	Right/Left	2/2	-
- Bilateral	Right > Left	11	-
	Left > Right	9	-
	Right = Left	10	-

Data are presented as mean ± standard deviation unless otherwise indicated, Faller is defined as having ≥ 1 fall in the past 12 months; *NRS* = Numerical Rating Scale; *NDI* = Neck Disability Index

### Standing conditions

#### Mean differences in postural sway

There were no significant interactions between group and condition for any postural sway variables (*p* > 0.05, ${\eta}_{p}^{2}$ = 0.001–0.007; Table 2). The main effects of group and condition were significant for all variables (group: *p* < 0.05, ${\eta}_{p}^{2}$ = 0.06–0.10; condition: *p* < 0.001, ${\eta}_{p}^{2}$ = 0.13–0.36; Table 2). The neck pain group demonstrated significantly greater postural sway for all variables compared with controls, regardless of condition (*p* < 0.05). The S-talk condition demonstrated significantly greater sway for all variables compared with the S and S-text conditions, regardless of group (*p* < 0.05). There were no significant differences between the S-text and S conditions for any variables (p > 0.05).

**Table 2:** Postural sway variables (mean ± SD) and main and interaction effects of group and condition

Variables	Condition	Group		Main effect^*^		Interaction^*^
				**Group**	**Condition**	**(Group x Condition)**
		**Neck pain**	**Controls**	***p*-value**	***p*-value**	***p*-value**
AP displacement (mm)	S	25.18 ± 7.68	21.30 ± 4.33	**0.047**	** < 0.001**	0.92
	S-text	25.27 ± 7.57	22.69 ± 5.70			
	S-talk	32.17 ± 15.08	28.30 ± 10.92			
ML displacement (mm)	S	19.63 ± 6.68	15.60 ± 4.74	**0.032**	** < 0.001**	0.94
	S-text	20.66 ± 12.39	17.84 ± 7.57			
	S-talk	35.11 ± 26.86	28.70 ± 18.15			
Sway area (mm^2^)	S	55.16 ± 28.96	37.30 ± 20.28	**0.012**	** < 0.001**	0.91
	S-text	67.64 ± 56.99	46.87 ± 32.07			
	S-talk	141.28 ± 122.39	119.77 ± 141.30			
Sway velocity (mm/s)	S	100.08 ± 15.77	94.60 ± 13.10	**0.039**	** < 0.001**	0.66
	S-text	101.75 ± 16.76	94.74 ± 12.84			
	S-talk	102.27 ± 15.98	97.53 ± 14.69			

Bold values indicate *p* < 0.05

^*^Log10-transformed data were used for analysis; S = Standing without using a smartphone; S-text = Standing while texting on a smartphone; S-talk = Standing while talking on a smartphone

#### Dual-task performance cost on postural sway

There were no significant interactions between group and condition for abs-DTC values of postural sway variables (*p* > 0.05, ${\eta}_{p}^{2}$ = 0.01–0.02; Table 3). The main effects of group and condition were significant for all variables (group: *p* < 0.05, ${\eta}_{p}^{2}$ = 0.07–0.09; condition: *p* < 0.01, ${\eta}_{p}^{2}$ = 0.13–0.30; Table 3). The neck pain group demonstrated significantly greater abs-DTC values for all variables compared with controls, regardless of condition (*p* < 0.05). The S-talk condition demonstrated significantly greater abs-DTC values for all variables than the S-text condition, regardless of group (*p* < 0.01).

**Table 3:** Abs-DTCs of postural sway variables and main and interaction effects of group and condition

Abs-DTC (%)	Condition	Group		Main effect^*^		Interaction^*^
				**Group**	**Condition**	**(Group x Condition)**
		**Neck pain**	**Controls**	***p*-value**	***p*-value**	***p*-value**
AP displacement	S-text	14.41 ± 25.77	7.61 ± 10.43	**0.040**	** < 0.001**	0.28
	S-talk	44.05 ± 44.31	32.98 ± 40.76			
ML displacement	S-text	45.64 ± 100.62	14.61 ± 40.74	**0.047**	** < 0.001**	0.52
	S-talk	130.91 ± 163.43	75.26 ± 105.12			
Sway area	S-text	78.20 ± 152.18	27.54 ± 38.91	**0.021**	** < 0.001**	0.27
	S-talk	237.62 ± 275.78	94.18 ± 97.30			
Sway velocity	S-text	3.32 ± 4.01	1.08 ± 1.54	**0.033**	** < 0.01**	0.43
	S-talk	3.44 ± 3.73	2.48 ± 3.15			

Bold values indicate *p* < 0.05

Data are presented as mean ± SD; ^*^Log10-transformed data were used for analysis; Abs-DTC = absolute dual-task performance cost; S = Standing without using a smartphone; S-text = Standing while texting on a smartphone; S-talk = Standing while talking on a smartphone

### Walking conditions

#### Mean differences in gait variables

There were no significant interactions between group and condition for any gait variables (*p* > 0.05, ${\eta}_{p}^{2}$ = 0.01–0.03; Table 4). The main effects of group and condition were significant for all variables (group: *p* < 0.05, ${\eta}_{p}^{2}$ = 0.07–0.09; condition: p < 0.001, ${\eta}_{p}^{2}$ = 0.38–0.68; Table 4). The neck pain group demonstrated significantly reduced gait speed, cadence, stride length, and step length, as well as increased stance time, swing time, and step width compared with controls, regardless of condition (*p* < 0.05). Regardless of group, the W-talk and W-watch conditions demonstrated significantly greater gait alterations for all variables compared with the W condition (*p* < 0.001). The W-talk condition demonstrated significantly greater gait alterations for all variables than the W-watch condition (*p* < 0.05).

**Table 4:** Gait variables (mean ± SD) and main and interaction effects of group and condition

Variables	Condition	Group		Main effect^*^		Interaction^*^
				**Group**	**Condition**	**(Group x Condition)**
		**Neck pain**	**Controls**	**p-value**	**p-value**	**p-value**
Gait speed (m/s)	W	1.00 ± 0.11	1.06 ± 0.13	**0.042**	** < 0.001**	0.74
	W-watch	0.87 ± 0.12	0.94 ± 0.14			
	W-talk	0.83 ± 0.13	0.88 ± 0.12			
Cadence (steps/min)	W	111.67 ± 8.32	114.19 ± 7.68	**0.026**	** < 0.001**	0.18
	W-watch	103.38 ± 9.36	109.22 ± 8.04			
	W-talk	100.64 ± 9.76	104.30 ± 7.84			
Stance time (s)	W	0.67 ± 0.06	0.64 ± 0.05	**0.040**	** < 0.001**	0.31
	W-watch	0.73 ± 0.08	0.68 ± 0.06			
	W-talk	0.75 ± 0.10	0.72 ± 0.07			
Swing time (s)	W	0.42 ± 0.02	0.41 ± 0.02	**0.043**	** < 0.001**	0.42
	W-watch	0.44 ± 0.04	0.42 ± 0.03			
	W-talk	0.45 ± 0.04	0.44 ± 0.03			
Stride length (m)	W	1.07 ± 0.09	1.11 ± 0.09	**0.033**	** < 0.001**	0.22
	W-watch	0.98 ± 0.08	1.04 ± 0.09			
	W-talk	0.97 ± 0.08	1.01 ± 0.09			
Step length (m)	W	0.54 ± 0.05	0.56 ± 0.04	**0.027**	** < 0.001**	0.39
	W-watch	0.49 ± 0.04	0.52 ± 0.04			
	W-talk	0.48 ± 0.04	0.50 ± 0.04			
Step width (m)	W	0.069 ± 0.03	0.058 ± 0.02	**0.032**	** < 0.001**	0.40
	W-watch	0.081 ± 0.03	0.068 ± 0.02			
	W-talk	0.078 ± 0.03	0.066 ± 0.02			

Bold values indicate *p* < 0.05

^*^ Log10-transformed data were used for analysis; W = Walking without using a smartphone; W-watch = Walking while watching a smartphone; W-talk = Walking while talking on a smartphone

#### Dual-task performance cost on gait variables

There were no significant interactions between group and condition for any gait variables (*p* > 0.05, ${\eta}_{p}^{2}$ = 0.00–0.02; Table 5). The main effects of group and condition were significant for all variables (group: *p* < 0.05, ${\eta}_{p}^{2}$ = 0.07–0.08; condition: *p* < 0.05, ${\eta}_{p}^{2}$ = 0.07–0.25; Table 5). The neck pain group demonstrated significantly higher abs-DTC values for all gait variables compared with controls, regardless of condition (*p* < 0.05). Regardless of group, the W-talk condition demonstrated significantly higher abs-DTC values for all gait variables, except step width, compared with the W-watch condition (*p* < 0.05). For step width, the W-talk condition demonstrated lower abs-DTC value than the W-watch condition (*p* < 0.001).

**Table 5:** Abs-DTC in gait variables and main and interaction effects of group and condition

Abs-DTC (%)	Condition	Group		Main effect^*^		Interaction^*^
				**Group**	**Condition**	**(Group x Condition)**
		**Neck pain**	**Controls**	**p-value**	**p-value**	**p-value**
Gait speed	W-watch	16.67 ± 10.19	10.98 ± 5.26	**0.034**	** < 0.01**	0.96
	W-talk	19.97 ± 10.37	16.96 ± 9.10			
Cadence	W-watch	8.21 ± 7.30	4.29 ± 3.79	**0.043**	** < 0.001**	0.47
	W-talk	11.04 ± 7.67	8.49 ± 7.00			
Stance time	W-watch	11.17 ± 12.97	4.80 ± 3.74	**0.040**	** < 0.001**	0.99
	W-talk	16.17 ± 12.97	10.85 ± 8.00			
Swing time	W-watch	5.95 ± 6.17	2.81 ± 2.54	**0.033**	** < 0.001**	0.61
	W-talk	9.70 ± 7.39	6.04 ± 5.88			
Stride length	W-watch	9.58 ± 5.18	6.81 ± 3.40	**0.046**	**0.025**	0.65
	W-talk	10.13 ± 4.43	9.07 ± 4.41			
Step length	W-watch	9.48 ± 5.57	6.72 ± 3.37	**0.035**	**0.037**	0.80
	W-talk	10.22 ± 4.53	8.91 ± 4.36			
Step width	W-watch	26.87 ± 21.35	14.17 ± 13.16	**0.044**	** < 0.001**	0.24
	W-talk	14.92 ± 18.54	9.69 ± 9.79			

Bold values indicate *p* < 0.05

Data are presented as mean ± SD; ^*^Log10-transformed data were used for analysis; abs-DTC = absolute dual-task performance cost; W = Walking without using a smartphone; W-watch = Walking while watching a smartphone; W-talk = Walking while talking on a smartphone

## Discussion

This study showed that older women with neck pain exhibited greater absolute performance costs in postural sway and gait across conditions compared to those without neck pain (as indicated by the abs-DTC group main effect), with no evidence of disproportionate dual-task interference, as a significant group × condition interaction was not observed. Additionally, smartphone use resulted in greater impairments in standing balance and walking performance compared to single-task baseline among older women. The results support altered cervical afferent input, which may contribute to changes in sensorimotor integration in individuals with neck pain. Effect sizes for between-group comparisons were small to medium (η^2^p = 0.06–0.10), suggesting that the observed differences could have potential clinical relevance [23].

Older women with neck pain demonstrated increased postural sway (i.e., ML and AP displacements, sway area, and sway velocity) across all conditions (standing alone and smartphone use) and higher abs-DTC values during smartphone use for all postural sway outcomes compared with controls. These findings may suggest overall impaired postural control in older women with neck pain. As no significant group-by-condition interaction was observed, the effect of task condition should be interpreted with caution. Our results are consistent with previous studies suggesting that patients with neck pain have impaired standing balance [3, 5]. Additionally, smartphone use during dual-task conditions, particularly talking on the phone, had a greater impact on postural control than texting or standing alone, irrespective of neck pain status. This aligns with previous studies demonstrating that phone use increases postural sway compared with standing alone, and that talking on a phone imposes greater postural demands than texting [12, 24]. Laatar et al. [12] found increased center of pressure (CoP) while phone talking and dialing compared to no phone use condition in both healthy younger and older adults. Talking produced higher CoP values than dialing. Likewise, Onofrei et al. [24] reported greater CoP path length and maximum CoP speed during both talking and texting conditions compared with a no-phone condition, with talking eliciting higher values than texting in healthy young adults. Notably, in this study, texting did not impair postural control relative to single-task standing, possibly reflecting compensatory strategies that prioritize postural stability over concurrent task performance. Taken together, the findings of the present study suggest that smartphone use may impose substantial cognitive and attentional demands, thereby increasing postural instability in older adults with neck pain.

Older women with neck pain demonstrated significant gait alterations, regardless of task conditions, compared with controls. Additionally, they exhibited higher abs-DTC values across dual-task conditions (watching and talking). These findings may suggest overall impaired gait performance in older women with neck pain. However, as no significant group-by-condition interaction was observed, the effect of task condition should be interpreted with caution. The alterations are consistent with previous studies reporting gait impairments under single- and dual-task conditions in older adults with neck pain [6, 25, 26]. Additionally, regardless of group, smartphone use (watching and talking) significantly altered gait performance (reduced gait speed, decreased cadence, prolonged stance and swing times, shorter stride and step length, and increased step width) compared to walking alone in older women, with talking eliciting greater gait alterations than watching. These findings are also consistent with previous findings, demonstrating that smartphone-related tasks, including texting, phone viewing, and question answering, adversely affect gait performance in both young and older adults [9, 13, 14]. Gait alteration during smartphone use may reflect adaptive strategies in older women that prioritize stability over locomotor efficiency under dual-task cognitive demands. Overall, the present findings support the notion that neck pain in older adults contributes to postural and gait disturbances beyond those associated with normal aging [4, 6], which are also evident during smartphone dual-task conditions. However, percentage-based DTC measures can be inflated when single-task (baseline) performance is low. Although dual-task cost in this study was expressed relative to baseline performance (%DTC) to reduce scaling effects, it remains mathematically coupled to baseline performance. Consequently, the higher abs-DTC values observed in older women with neck pain should be interpreted with caution, as these differences may be partly attributable to poorer baseline performance rather than indicating an independent vulnerability to dual-task conditions.

Several explanations may underlie postural and gait disturbances observed during smartphone use, particularly during phone conversation. Increased postural sway and gait adaptations may result from divided attention toward a secondary task, particularly during talking. Conversational speech generally requires greater attentional resources due to its interactive, real-time nature and cognitive-linguistic demands for verbal responses compared with texting or passive viewing on a smartphone [27, 28]. Texting involves combined cognitive, visual, and motor demands, whereas watching primarily involves visual demands [29]. The relatively fixed gaze adopted during these tasks may provide a stable visual reference, potentially reducing head and neck sway through visual fixation and activation of the vestibulo-ocular reflex [30]. Specifically, in older women with neck pain, neck pain may be exacerbated by compounded cervical afferent deficits and cognitive-motor interference. Abnormal afferent input from the neck induces a sensory mismatch with other inputs within the central nervous system [1, 2]. This mismatch, when combined with the increased attentional demands of concurrent smartphone use, may compromise the ability to integrate sensory information and allocate attention between gait and cognitive tasks. Additionally, walking while using a smartphone disrupts natural head-neck alignment, which may place greater demands on active head-neck control, potentially increasing aberrant cervical afferent input. Additional physical demand, such as reduced arm swing, may also play an important role in modifying gait during smartphone use. These factors may contribute to greater impairments in postural and gait stability during smartphone use in older adults with neck pain. Nevertheless, these proposed mechanisms remain speculative and should be explored in future studies.

A gait speed below 1.0 m/s is considered indicative of an increased risk of falls [31]. Outdoor walking at a usual pace averages approximately 1.31 m/s [32]. It has been suggested that the assumed 1.31 m/s speed for outdoor walking is insufficient for mobility-limited older adults to cross a street safely at a comfortable walking speed [33, 34]. Thus, older adults, particularly those with neck pain, may be at increased risk of falling and encountering environmental hazards when walking while using smartphones. These findings underscore the importance of increasing awareness regarding smartphone use during standing and walking, as well as implementing prevention and rehabilitation programs targeting postural control and gait performance to enhance safety and mobility in older populations.

Some limitations of the study should be acknowledged. Only females were included in the study, which may limit the generalizability of our findings to males. Some potential confounding factors, such as cognitive function and physical activity levels, were not specifically controlled. Kinematic data were not recorded during the standing balance conditions, limiting the ability to definitively confirm standardization of the base of support and potentially introducing variability in postural demands across participants. Variations in arm position and smartphone viewing distance may also have mechanically influenced CoP displacement. However, these factors were intentionally not strictly standardized to allow for self-selected postures that better reflect naturalistic smartphone use and reduce the artificial postural tension induced by rigid experimental constraints. Task prioritization was also not examined, which may have further influenced dual-task performance. Furthermore, conducting multiple separate ANOVAs across outcomes may increase the risk of Type I error. As abs-DTC is mathematically dependent on single-task performance, group differences in abs-DTC cannot be interpreted independently of baseline differences. Future studies should consider baseline-adjusted analytical approaches, such as covariate-controlled models, to better distinguish dual-task effects from underlying differences in single-task performance. Further investigation is warranted to clarify the association between postural and gait changes and fall risk in older adults with neck pain. Future studies should address potential confounding factors to better determine the specific effects of neck pain and incorporate standardized cognitive assessments to account for the contribution of cognitive function to dual-task performance. Exploring the mechanisms underlying altered sensorimotor integration and cognitive-motor interactions may help identify targeted strategies to enhance postural and gait stability during dual-task conditions in older adults with neck pain.

## Conclusion

Older women with neck pain demonstrated greater impairments in postural control and gait performance across all conditions, including smartphone dual-task conditions, compared with controls. Dual-task smartphone use resulted in increased postural sway and greater gait alterations across groups. The alterations were greater during talking, whether standing or walking, than during texting while standing or watching while walking. These findings have implications for awareness of smartphone use in older women.

## Data Availability

The datasets used and/or analyzed during the current study are available from the corresponding author on reasonable request.

## Abbreviations

abs-DTC: Absolute dual-task performance cost
AP displacement: Antero-posterior displacement
BMI: Body mass index
CoP: Center of pressure
DTC: Dual-task performance cost
ML displacement: Medio-lateral displacement
NDI: Neck Disability Index
NRS: Numerical Rating Scale
${\eta}_{p}^{2}$: Partial eta squared
S: Standing with no smartphone use
S-talk: Standing while talking on a smartphone
S-text: Standing while texting on a smartphone
W: Walking with no smartphone
W-talk: Walking while talking on a smartphone
W-watch: Walking while watching on a smartphone
